# Effects of co-exposure to CS_2_ and noise on hearing and balance in rats: continuous versus intermittent CS_2_ exposures

**DOI:** 10.1186/s12995-020-00260-5

**Published:** 2020-05-11

**Authors:** Monique Chalansonnet, Maria Carreres-Pons, Thomas Venet, Aurélie Thomas, Lise Merlen, Stéphane Boucard, Frédéric Cosnier, Hervé Nunge, Elodie Bonfanti, Jordi Llorens, Pierre Campo, Benoît Pouyatos

**Affiliations:** 1 Institut National de Recherche et de Sécurité, Rue du Morvan, CS 60027, Cedex, 54519 Vandœuvre, France; 2 Departament de Ciències Fisiològiques and Institute of Neurosciences, Universitat de Barcelona, 08907 L’Hospitalet de Llobregat, Catalonia Spain; 3 Institut d’Investigació Biomèdica de Bellvitge (IDIBELL), 08907 L’Hospitalet de Llobregat, Catalonia Spain; 4 DevAH EA 3450 – Développement, Adaptation et Handicap, Régulations cardio-respiratoires et de la motricité-Université de Lorraine, 54500 Vandœuvre, France

**Keywords:** Carbon disulfide, Low-frequency noise, Cochlea, Vestibule, Co-exposure, Rat

## Abstract

**Background:**

Carbon disulfide (CS_2_) exacerbates the effect of noise on hearing, and disrupts the vestibular system. The goal of this study was to determine whether these effects are also observed with intermittent CS_2_ exposure.

**Methods:**

Rats were exposed for 4 weeks (5 days/week, 6 h/day) to a band noise at 106 dB SPL either alone or combined with continuous (63 ppm or 250 ppm) or intermittent (15 min/h or 2 × 15 min/h at 250 ppm) CS_2_. Hearing function was assessed by measuring distortion product otoacoustic emissions (DPOAEs); balance was monitored based on the vestibulo-ocular reflex (VOR). Functional measurements were performed before, at the end of exposure and 4 weeks later. Histological analyses of the inner ear were also performed following exposure and after the 4-week recovery period.

**Results:**

The results obtained here confirmed that CS_2_ exposure exerts two differential temporary effects on hearing: (1) it attenuates the noise-induced DPOAE decrease below 6 kHz probably through action on the middle ear reflex when exposure lasts 15 min per hour, and (2) continuous exposure to 250 ppm for 6 h extends the frequency range affected by noise up to 9.6 kHz (instead of 6 kHz with noise alone). With regard to balance, the VOR was reversibly disrupted at the two highest doses of CS_2_ (2 × 15 min/h and continuous 250 ppm). No morphological alterations to the inner ear were observed.

**Conclusion:**

These results reveal that short periods of CS_2_ exposure can alter the sensitivity of the cochlea to noise at a dose equivalent to only 10 times the short-term occupational limit value, and intermittent exposure to CS_2_ (2 × 15 min/h) can alter the function of the vestibular system.

## Background

Carbon disulfide (CS_2_) is a lipophilic, volatile, inflammable solvent, used in large quantities in the production of viscose rayon fibres and cellophane films [1]. Upon inhalation, CS_2_ rapidly reaches the bloodstream and is distributed throughout the body, where, due to its lipophilic nature, it preferentially accumulates in organs with a high fat content, including the brain and the liver.

Cases of vascular complications [2, 3] and damage to endocrine structures [4] have been reported in workers exposed to CS_2_. However, the most common adverse effect of CS_2_ described in the literature is the emergence of neurofilamentous axonopathies [5–7], which can affect both sensory and motor neurons [8, 9]. As a result, CS_2_ is classified as a neurotoxic compound.

To better understand its toxicity, CS_2_ has been extensively studied in rats, where an abnormal accumulation of neurofilaments in the long axons of the peripheral (PNS) and central nervous system (CNS) has been observed [7, 10–12]. At the level of the auditory system, several studies examining auditory brainstem responses reported conduction dysfunction [13–15].

We previously studied the effects of a four-week exposure to CS_2_ on both the peripheral vestibular and auditory systems in rats. No histological damage was observed within the inner ear, suggesting that CS_2_ is neither vestibulotoxic nor cochleotoxic [16, 17]. However, CS_2_ exposure did induce a temporary functional impairment of the vestibular system (via a possible action on the CNS, which was augmented by noise [16]). CS_2_ also exacerbated the transient effects of noise on the hearing system by extending auditory losses toward high frequencies [17].

Currently, the French occupational exposure limit (OEL) and the European indicative occupational exposure limit value (IOELV) for CS_2_ are both 5 ppm. In United States, the permissible exposure level (PEL), defined by the Occupational Safety and Health Administration, is 20 ppm. Based on numerous epidemiological studies, experts recommend even lower limit values, ranging between 1 and 10 ppm [18, 19]. CS_2_ concentrations in the workplace are also regulated by a short-term exposure limit **(STEL)**: 15-min spot exposures must not exceed 25 ppm in France or 30 ppm in the United States, and workers must not be exposed to these concentrations more than four times per day, with at least 60 min between exposure periods. Despite these limitations, industrial viscose production, daily exposures of approximately 40 ppm have been reported in the literature [20, 21].

In industry, CS_2_ exposure is frequently associated with noisy work environments. Several epidemiological studies [22–24] show that there is a greater incidence of hearing loss in workers co-exposed CS_2_ (8.6 to 30 ppm) and noise (> 85 dB(A)) than in worker exposed to noise alone. Studies by the Sulkowski’s group [25, 26] also suggest an unfavorable interaction between high doses of CS_2_ (> 90 ppm) and noise (> 86 dB(A)) on the balance function. According to these studies, the lowest observed adverse effect (LOAEL) on hearing, when noise above 85 dB(A) is also present, can be estimated at about 10 ppm.

The combined impact of CS_2_ and noise on the vestibular organ might seem surprising, but several authors have shown that noise, and more specifically low-frequency noise, is detected by, and perturbs, the vestibule [27, 28]. Therefore, both solvents and noise, alone or in combination, may affect balance in addition to impairing hearing.

Although the average dose of acoustic energy and mean concentration of CS_2_ play a significant role in the occupational risk associated with exposure to each or both of these factors, the pattern of exposure may have an even more profound impact. With regard to noise, it is now well established that impulse noises are much more damaging than continuous noises at equivalent levels [29, 30]. In contrast, how the temporal pattern of exposure to chemicals influences their toxic effects has only rarely been investigated [29]. Nevertheless, given that chemical concentrations in the air are often related to specific tasks in occupational settings and may therefore be very irregular during the workday, it is essential to determine how intermittent exposure compares to constant exposure in terms of adverse biological effects.

The need for this comparison is especially pressing in the case of solvents, which, because of their lipophilic properties, can persist in tissues, even after they have been eliminated from the blood stream. Indeed, 40% of blood CS_2_ is lost within 6 min, whereas CS_2_ levels in tissues continue to increase after the end of exposure [31, 32].

For these reasons, the main purpose of the current animal-based laboratory investigation was to study how co-exposure to CS_2_ with different temporal patterns and low-frequency noise (L_EX,8h_ = 105 dB SPL, band-pass filtered over three octave bands centered at 0.5, 1 and 2 kHz) affected hearing and balance. Two groups of rats were exposed 6 h/day to noise and to the equivalent total daily dose of CS_2_ but with different temporal patterns: 63 ppm continuously vs. 250 ppm for 15 min every hour (10 times the French STEL). Three additional conditions were included in the experimental design: a high continuous CS_2_ dose (250 ppm i.e., 40 times the European IOELV, considering that the exposure duration is 6 h and not 8 h), intermittent CS_2_ exposure with 15 min of CS_2_ every 30 min, and a non-exposed group. These last three conditions were included in the study to determine, if possible, at what interval intermittent CS_2_ exposure becomes equivalent to continuous exposure in terms of adverse effects induced.

The impact of the exposure pattern on hearing was assessed using distortion product oto-acoustic emissions (DPOAEs) which reflect outer hair cell (OHC) motility [33]. Vestibular function was estimated by measuring post-rotary nystagmus (PRN) [16, 34–36], which is a good correlate of the vestibulo-ocular reflex (VOR) [37].

Functional investigations were complemented by morphological analyses of the inner ear and by monitoring blood CS_2_ concentrations and urine 2-thiothiazolidine-4-carboxylic acid (TTCA) concentrations. TTCA is a urinary CS_2_ metabolite considered to be a good biological exposure indicator [38].

The results obtained are discussed with regard to current occupational exposure levels (OEL and STEL), which should regulate the risks encountered by all workers, including those who are co-exposed to several risk factors.

## Methods

### Animals

While conducting the research described in this article, investigators adhered to the Guide for Care and Use of Laboratory Animals promulgated by the European parliament and council [39]. The study protocol was approved by the local ethics committee and by the French Ministry of Education and Research (APAFIS#3950–201,602,051 1,372,481). The animal facility is fully accredited by the French Ministry of Agriculture (authorization No. D 54–547-10). A total of 165 adult female Long Evans rats weighing 240 g on average were used in the current study. Eight-week old rats were purchased from Janvier labs (Le Genest St Isle, St Berthevin, 53,941, France); they were 18 weeks old at the start of the exposure protocol. The animals were housed two per cage (1032 cm^2^ × 20 cm height) on irradiated cellulose BCell8 bedding (ANIBED, Pontvallain, France). A 12:12 h light:dark cycle (07:30–19:30) was maintained in the facility, room temperature was 22 ± 2 °C, and relative humidity 55 ± 10%. Food and tap water were available ad libitum, except during exposure. The background noise level in the animal facility was around 43.5 dB(A). Animals were weighed weekly and monitored for overall toxicity, as defined in [40].

### Protocol

**Fig. 1:**
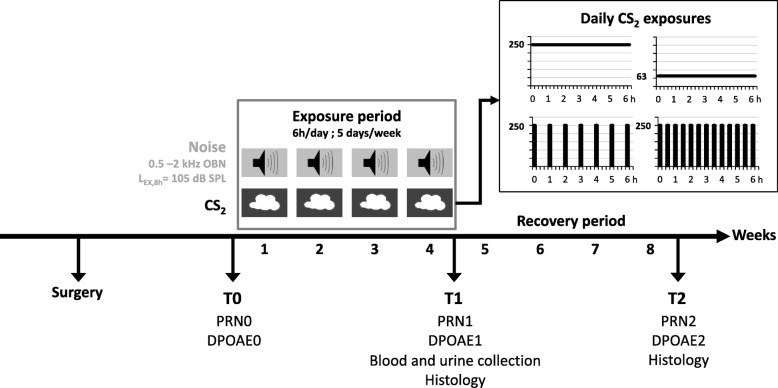
Experimental protocol. Rats were exposed to carbon disulfide (CS_2_) and noise for 6 h/day, 5 days/week, over 4 weeks. The pink noise level was 106 dB SPL (L_EX,8h_ = 105 dB SPL) and the spectrum was a band-pass filtered over three octave bands centered at 0.5, 1 and 2 kHz. CS_2_ exposure was continuous (63 or 250 ppm) or intermittent (1 × 15 min/h or 2 × 15 min/h at 250 ppm CS_2_). Distortion product otoacoustic emissions were used to test hearing prior to exposure (T0: DPOAE0), following the exposure period (T1: DPOAE1), and after a 4-week recovery post-exposure period (T2: DPOAE2). Vestibular function was assessed using post-rotary nystagmus measurements prior to exposure (T0: PRN0), at the end of the exposure period (T1: PRN1), and after the 4-week recovery period (T2: PRN2). Blood and urine samples were collected at T1. Histological analyses were performed at T1 and T2

An overview of the experimental protocol is shown in Fig. 1.

Animals were exposed to a low-frequency noise with or without concomitant CS_2_. The noise was a continuous pink noise at 106 dB SPL (L_EX,8h_ = 105 dB SPL) band-pass filtered over three octave bands centered at 0.5, 1 and 2 kHz. Solvent exposure was either continuous (63 or 250 ppm) or intermittent (1 × 15 min/h or 2 × 15 min/h at 250 ppm). Exposure lasted 6 h per day for 5 consecutive days over 4 weeks. The number of controls and exposed animals used in each condition are listed in Table 1. In line with the reduce/replace/refine principles, several experimental techniques were performed on samples from individual animals.

**Table 1:** Numbers of animals used in this study for each experimental condition

**Method**	PRN	DPOAE	Light microscopy		SEM	**Total number of animals per group**
**Time-points**	T0/T1/T2	T0/T1/T2	T1	T2	T2	
**Controls**	36	35	10	10	10	**71**
**Noise**	12	12	5	6	5	**24**
**63 ppm CS**_**2**_** + Noise**	7	8	–	4	4	**15**
**1 × 15 min 250 ppm CS**_**2**_** + Noise**	11	9	5	5	5	**20**
**2 × 15 min 250 ppm CS**_**2**_** + Noise**	7	11	–	–	–	**18**
**250 ppm CS**_**2**_** + Noise**	13	11	4	5	5	**24**

*CS*_*2*_ carbon disulfide, *DPOAE* Distorsion Product Oto-Acoustic Emission, *PRN* post-rotatory nystagmus, *T0* prior to exposure, *T1* immediately following exposure, *T2* 4 weeks post-exposure, *SEM* scanning electron microscopy. NB: As several analytical techniques were performed on samples from individual animals, the number of animals indicated in the right-most column does not correspond to the sum of the five other columns

The vestibular function was tested based on PRN measurements, and the rats’ hearing was measured using cubic DPOAEs. These tests were performed prior to (T0), immediately after exposure (T1), and 4 weeks post-exposure (T2). Blood samples were collected from rats co-exposed to 63 ppm CS_2_ and noise, rats co-exposed to noise and 250 ppm CS_2_ for1x15 min, and from control animals at the end of the exposure period to determine circulating CS_2_ concentrations. Urine samples were also collected from these animals to analyze TTCA concentrations. Twenty-four animals were sacrificed at the end of the exposure period to perform morphological analyses (Table 1). The remaining animals were left in their home cage to recover for 4 weeks. At the end of the experiment, all animals were euthanized to collect the cochleae and the vestibular sensory epithelia for morphological observation.

### Anesthesia

Rats were anesthetized by a single injection of a mixture of ketamine/xylazine (45/5 mg/kg i.p.) before performing surgery and DPOAE tests. Body temperature was continuously monitored throughout the procedure using a rectal probe connected to a system maintaining body temperature between 34 and 36 °C. For blood collection, rats were anesthetized with 3% isoflurane; for tissue collection, deep anesthesia was induced with a mixture of ketamine/xylazine (75/5 mg/kg*, i.p.*).

### Distortion product Otoacoustic emissions

DPOAEs were recorded at T0 (DPOAE0), T1 (DPOAE1) and T2 (DPOAE2). The device and methodology used to record DPOAEs are detailed in [29]. Briefly, six pairs of primary tones (f1-f2): (3–3.6), (4–4.8), (5–6), (8–9.6), (14.6–17.52), and (21.2–25.44) kHz were delivered to the left ear. The f1 to f2 ratio for these primaries was always 1.2 [41], and the level difference (L1 – L2) was 14 dB. To simplify the presentation of results hereafter, each pair of primaries will be indicated only by the f2.

At the end of the exposure, hearing variations were calculated as [DPOAE1 – DPOAE0] – K1. The constant K1 was taken as the average [DPOAE1 – DPOAE0] determined for the relevant control group. After the 4-week recovery period, variations were computed as [DPOAE2 – DPOAE0] – K2. Where the constant K2 was taken as the average of [DPOAE2 – DPOAE0] calculated for the relevant control group.

### Surgical procedure and habituation

To immobilize the animal during the PRN procedure, a screw nut was placed on the skull. The surgical procedure is detailed in [16] and was performed 2 weeks before the first measurements. Briefly, the skin over the vertex was incised and the skull devitalized with silver nitrate, after clearing it of connective tissue. A screw nut was affixed to the bone using cyanoacrylate and embedded in dental cement (Taab 2000®).

One week post-surgery, the animals were progressively habituated to the PRN procedure.

### Post-rotatory nystagmus test

PRN tests were performed as detailed in [16]. Briefly, the home-made test apparatus consisted of a dark circular arena (102.5 cm diameter) containing a horizontal turntable. The rat was rotated around a dorso-ventral axis located approximately between its shoulder blades. During rotation (40 s at 90 °/s), the rat’s head was held at a fixed position with a screw placed on a metal bar in the center of the turntable. After rotation, horizontal movement of the left pupil was recorded in the dark using a RK-826PCI eye tracker (240 Hz sampling rate; ISCAN, Inc. Twenty-one Cabot Road Woburn, MA 01801 USA). The VOR parameters recorded were the number and duration of saccades.

### Carbon disulfide exposure

During exposure, all rats were housed in individual cells within an inhalation chamber designed to sustain a dynamic, adjustable airflow (5–6 m^3^.h^− 1^). The chambers were maintained at a negative pressure of no more than 3 mm H_2_O. Input air was filtered and conditioned to a temperature of 22 ± 1 °C and relative humidity of 55 ± 10%.

CS_2_ was generated using a thermoregulated glass streamer. The solvent was delivered by a pump and instantaneously vaporized upon contact with the heated surface of the glass streamer. The vapor was carried forward with an additional airflow through the streamer, into the main air inlet pipe of the exposure chambers. Rats were exposed continuously to 63 or 250 ppm of CS_2_, or to 250-ppm CS_2_ for 15 min per hour or 2 × 15 min per hour for 6 h per day on 5 consecutive days over 4 weeks. Control animals (*n* = 71) were always ventilated with fresh air.

The exposure concentrations were verified using samples of atmosphere collected in the chambers using glass tubes packed with Carboxen 1000 40/60 mesh (Supelco). CS_2_ was desorbed from the adsorbent with dichloromethane (DCM). Methyl-ethyl-ketone (MEK) was added as an internal standard (IS) and samples were analyzed on a gas chromatograph/mass spectrometer (GCMS-QP2010 Ultra, Shimadzu). CS_2_ samples were assayed on a 30 m × 0.25 mm (1 μm film thickness) Rtx-1701 column (with integra-guard) (Restek), using helium as the carrier gas at a linear velocity of 45 cm/s. The column temperature program was 40 °C for 3 min followed by an increase to 100 °C at a rate of 20 °C/min. The sample (1 μL) was injected in split mode with a split ratio of 1/30. The temperatures for the injection port, transfer line and ion source were set to 240 °C, 250 °C and 200 °C, respectively. The MS was operated by electron ionization (70 eV) in selected ion monitoring mode, tracking ions 43 (for IS) and 76 (for CS_2_). These analyses allowed daily calibrations to be performed. During exposure, a benchtop mass spectrometer with yttrium-iridium filament (OmniStar GSD 320 O2, Pfeiffer vacuum) was also used to continuously monitor the stability of vapor generation throughout the exposure period.

### Noise exposure

Device, methodology and type of noise were detailed in [17]. Briefly, the noise exposure was performed within the inhalation chambers. Rats were housed in individual cells inside the chambers. The noise was a filtered pink noise with a band-pass filtered over three octave bands centered at 0.5, 1 and 2 kHz. This noise corresponds to the low-frequency region of the rat’s hearing range. As rats show a low sensitivity in this frequency range, the exposure level was set to 106 dB SPL and was maintained 6 h per day.

### Blood sample collection and blood analysis

Blood was collected from the tail vein in eight rats exposed to 63 ppm and seven rats exposed to 250 ppm CS_2_ for 1 × 15 min/h. Samples (~ 0.8 mL) were dispensed into 2-mL heparinized vials and frozen at − 20 °C until analysis.

Blood samples were acidified with 250 μL HCl (2%) to release bound CS_2_ and stirred for 10 min after the addition of 10 μL of an IS (1.5 g/L MEK in DCM) [42]. Samples were extracted with 500 μL DCM. After shaking for 30 min and centrifugation (3220 g at − 4 °C for 20 min), the DCM layer was recovered and analyzed in the same chromatographic conditions as described for atmospheric monitoring. In these conditions, the assay was linear between the limit of quantification (= 0.3 μg) and 15 μg CS_2_; the limit of detection for the method was around 0.1 μg; accuracy was less than − 12.4%; and precision was close to 15%.

### Urine collection and TTCA analysis

At the end of exposure, the rats from which blood had been collected were placed in individual metabolic-type stainless steel cages with free access to food and water from 3:00 pm to 9:00 am the following day. During this 18-h period, urine was collected and refrigerated by a cooling system surrounding the collection tubes. Urine samples were frozen immediately after collection and stored at − 20 °C until the day of analysis. The TTCA concentration in urine was determined using an automated column-switching by high-performance liquid chromatography, as previously described [43]. Dilute urine was purified on an anion-exchange column, the fraction of interest was transferred for isocratic analysis on a cyano-amino column. TTCA was detected by measuring UV absorption at 275 nm. In these conditions, the assay was linear between the limit of quantification (0.15 mg/L) and 50 mg/L; the limit of detection was below 0.05 mg/L; recovery exceeded 95% and within- and between-day precision were less than 1 and 4%, respectively.

### Light microscopy analyses

Immediately following exposure or after the 4-week post-exposure recovery period, the left cochleae and the right vestibule were collected from deeply anesthetized animals.

The round and oval windows of the cochlea were opened and 2.5% glutaraldehyde in 0.2 M cacodylate buffer was injected into the labyrinth. Cochleae were immersed in the same fixative for 15 days at 4 °C, then rinsed in cacodylate buffer and post-fixed for 1 h in 1% osmium tetroxide. Cochleae were drilled, decalcified and dehydrated in graded concentrations of ethanol up to 100%, then placed in (50/50) resin/propylene oxide, followed by (75/25) resin/propylene oxide, and finally embedded in 100% resin (Epon/Araldite).

Vestibules were fixed for 7 days by immersion in 2.5% glutaraldehyde in 0.2 M cacodylate buffer at 4 °C before dissection under a light binocular microscope. Epithelia and Scarpa’s ganglion were rinsed in cacodylate buffer, post-fixed for 1 h in 1% osmium tetroxide and dehydrated in graded concentrations of ethanol up to 100%. They were then embedded in resin (Epon/Araldite).

After polymerization at 60 °C, embedded cochlear and vestibular specimens were cut to produce semi-thin sections (2.5 μm) which were stained with cresyl violet and observed under an Olympus BX41 optical microscope.

### Scanning electron microscopy

Cochlear specimens were prepared following intracardiac fixation (2.5% glutaraldehyde in 0.2 M cacodylate buffer), whereas vestibular fixation was achieved by immersion in the same fixative solution. Consequently, scanning electron microscopy observations of these two tissues required the use of different animals.

Cochleae or vestibules were removed from the skull, perfused and immersed in the fixative solution at 4 °C for 24 h or 48 h, respectively. Tissues were then rinsed and immersed in 1% osmium tetroxide, 0.2 M cacodylate buffer for 1 h. Cochleae were drilled and dissected to expose the organ of Corti. Vestibules were dissected under a binocular microscope to isolate only the saccules, utricles and the three crista ampullaris.

After dehydration in graded ethanol solutions up to 100% ethanol, samples were placed in a critical-point dryer and dried using liquid CO_2_, before sputter-coating with gold. Samples were observed with a JEOL7400F scanning electron microscope.

### Statistical analyses

Statistical analyses were performed using Prism V7.03 (GraphPad. Software Inc., La Jolla, CA). The results are expressed as follows: F (dfb, dfr) = F-ratio; p = *p* value, in which dfb is the number of degrees of freedom between groups, and dfr is the number of residual degrees of freedom. The threshold for statistical significance was set to 95%. Data are expressed as mean ± SEM.

A one-way ANOVA was used to assess the statistical significance of variations in DPOAE amplitudes between exposed and control rats at each frequency. Holm-Sidak’s post-hoc test was applied when performing between-group comparisons.

PRN measurements and the weight of the animals were statistically analyzed by applying repeated-measure two-way ANOVAs with “treatment” as the between-subject factor and “time” as the within-subject factor. Bonferroni’s post-hoc comparisons were performed for each treatment group versus T0 (for PRN) or versus the control group (for weight). PRN data are expressed as a percentage of baseline (T0) values.

A t-test was used to analyze the statistical significance of differences in TTCA concentration and CS_2_ blood concentration between two groups following exposure.

## Results

### General health

Over the 4 weeks of exposure, the weights of the animals co-exposed to continuous CS_2_ at 250 ppm and noise remained stable between T0 and T1, whereas controls, noise-exposed animals, animals co-exposed to continuous CS_2_ at 63 ppm and noise and animals intermittently co-exposed to CS_2_ and noise gained some significant weight. The “treatment” x “time” interaction was F(10, 264) = 7.172; *p* < 0.0001. The only significant difference in weight at T1 was between the CS_2_ 250 ppm + noise group and controls (*p* = 0.0331, Bonferroni). The 250-ppm CS_2_ concentration delivered continuously can therefore be considered as the threshold for toxicity. However, according to the standard definition presented in [40], all rats remained in good health throughout the experiment. This effect on the weight gain was temporary since, there were no significant differences in weight between treatment groups at T2 (*p* > 0.99; Bonferroni).

### Metabolism

**Fig. 2:**
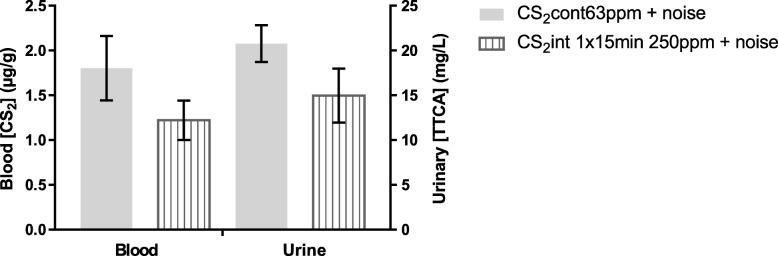
Blood CS_2_ and urinary TTCA concentrations measured at T1 were not significantly different between animals exposed to the same overall dose of CS_2_: 63 ppm continuous and 250 ppm 1 × 15 min/h. Right y-axis: urinary TTCA concentration (mg/L); left y-axis: blood CS_2_ concentration (μg/g). CS_2_: carbon disulfide, TTCA: 2-Thio-1,3-thiazolidine-4-carboxylic acid. Values correspond to mean ± SEM (*n* = 8/group)

Figure 2 shows blood CS_2_ and TTCA concentrations measured at the end of exposure in animals exposed to the same overall dose of CS_2_ (63 ppm continuous and 250 ppm 1 × 15 min/h). Although the urinary TTCA and blood CS_2_ concentrations measured following the intermittent exposure were slightly lower than those following the continuous exposure, the difference was not significant (*p* = 0.1282 and *p* = 0.2065, respectively).

### Hearing test

DPOAEs were acquired as DPgrams from 3.6 to 25.44 kHz. The DPOAE variations measured at all the investigated frequencies are displayed in Suppl. Figure 1. In the absence of CS_2_ co-exposure, the continuous noise induced a clear decrease of DPOAEs between 3.6 and 6 kHz at T1, which reached a maximum of 13 dB at 4.8 kHz.

**Fig. 3:**
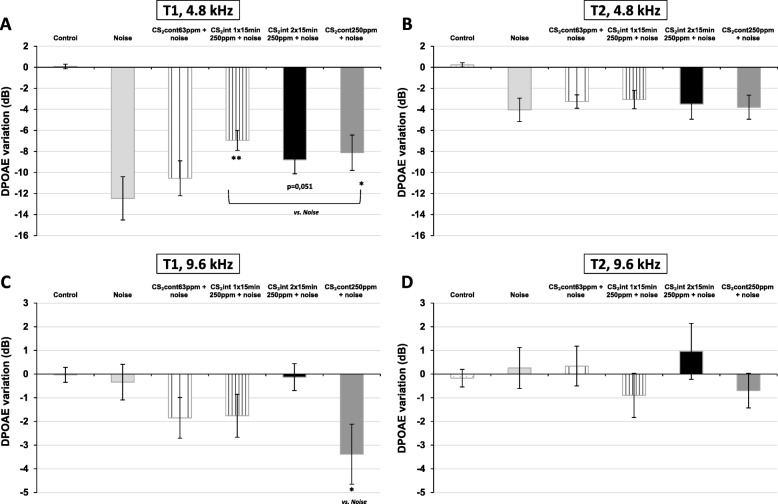
DPOAE variations for the five experimental groups at 4.8 kHz (upper panels) and 9.6 kHz (lower panels) following exposure (T1; left panels) and after the recovery period (T2; right panels). The method used to calculate DPOAE variations is detailed in the methods section. Values correspond to mean ± SEM. * *p* < 0.05, ** *p* < 0.01, compared to the mean for the group exposed to noise alone, Holm-Sidak test

To facilitate the comparisons between the numerous experimental groups, only the DPOAE variations measured at 4.8 and 9.6 kHz are shown in Fig. 3. At T1, all exposure conditions had a significant effect on DPOAE amplitude variations at 4.8 kHz [F (5, 92) = 32.15; *p* < 0.0001] and 9.6 kHz [F (5, 91) = 3.84; *p* = 0.0034]. When compared to the effect of noise alone, co-exposure to CS_2_ at 250 ppm for 1 × 15 min/h and noise induced a smaller decrease in DPOAE (*p* **=** 0.007 vs. noise, Holm-Sidak) at the frequency of 4.8 kHz. In contrast, the group co-exposed to CS_2_ 63 ppm and noise, which received the same overall dose of CS_2_ but in a continuous pattern, showed no significant difference in DPOAE (*p* = 0.28 vs. noise, Holm-Sidak). Decreases in DPOAE variation were also observed with the two other groups: CS_2_ 250 ppm 2 × 15 min/h + noise (*p* = 0.051 vs. noise, Holm-Sidak) and CS_2_ 250 ppm administered continuously (*p* = 0.026 vs. noise, Holm-Sidak). After the 4-week recovery period (T2), the DPOAE variations were identical in all exposed groups (*p* > 0.98 vs. noise, Holm-Sidak; Fig. 3b) but did not returned to control levels (*p* < 0.0011 vs. controls, Holm-Sidak).

At T1, no effect of noise alone was recorded at 9.6 kHz, whereas co-exposure to noise and continuous CS_2_ at 250 ppm resulted in a significant decrease in hearing (− 3.4 dB) at this frequency (*p* = 0.026 vs. noise, Holm-Sidak). In contrast, in animals exposed to lower doses of CS_2_ (continuous 63 ppm, 1 × 15 min/h at 250 ppm and 2 × 15 min/h at 250 ppm), no change was observed when compared to the DPOAE variation obtained in the noise-only group (*p* = 0.59, p = 0.59, *p* = 0.92, respectively, Holm-Sidak). After the 4-week recovery period (T2), the DPOAE levels had returned to control levels in all experimental groups (*p* > 0.99 vs. controls, Holm-Sidak; Fig. 3d).

### Post-rotatory nystagmus

**Fig. 4:**
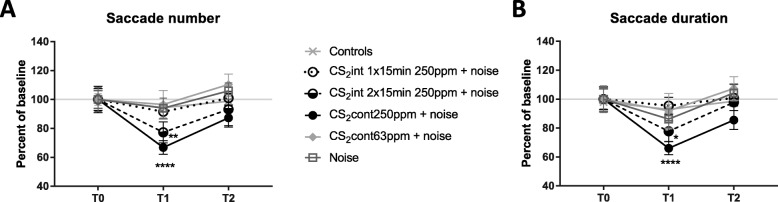
Post-rotatory nystagmus is affected by co-exposure to noise and CS_2_. Saccade number (**a**) and duration (**b**) measured for each experimental group at T0, T1 and T2 expressed as percent of baseline. Data correspond to mean ± SEM. * *p* < 0.05, ** *p* < 0.01, **** *p* < 0.0001, significantly different from the T0 mean, Bonferroni’s post-hoc comparisons

Figure 4 shows the average saccade number (A) and duration (B) measured for the different experimental groups before (T0), immediately after exposure (T1) and following the recovery period (T2). The interaction between “treatment” and “time” was significant for both parameters: saccade number [F (10, 158) = 2.534; *p* = 0.0074] and duration [F (10, 158) = 1.955; *p* = 0.0417].

These two parameters remained stable at all time-points in controls, animals exposed to noise alone, animals co-exposed to noise + 63 ppm of CS_2_ and animals co-exposed to noise + 1 × 15 min/h of CS_2_ at 250 ppm.

In contrast, at T1, the animals from the two remaining groups, i.e. noise + 2 × 15 min/h of 250 ppm CS_2_ and noise + continuous 250 ppm-CS_2_, showed a significant decrease in saccade numbers (− 23%, *p* = 0.0069 and − 33%, *p* < 0.0001 vs. T0, respectively, Bonferroni) and saccade duration (− 22%, *p* = 0.0269 and − 34%, *p* < 0.0001 vs. T0, respectively, Bonferroni). Following the 4-week recovery period, both parameters measured in all exposed groups had returned to baseline values (*p* > 0.05 vs. T0, Bonferroni). However, the values obtained at T2 in animals co-exposed to noise + continuous 250 ppm-CS_2_ tended to remain lower than those from the other experimental groups, although the difference compared to its T0 values was just below statistical significance (number: *p* = 0.0511; duration: *p* = 0.0554; Bonferroni).

### Histological analyses

**Fig. 5:**
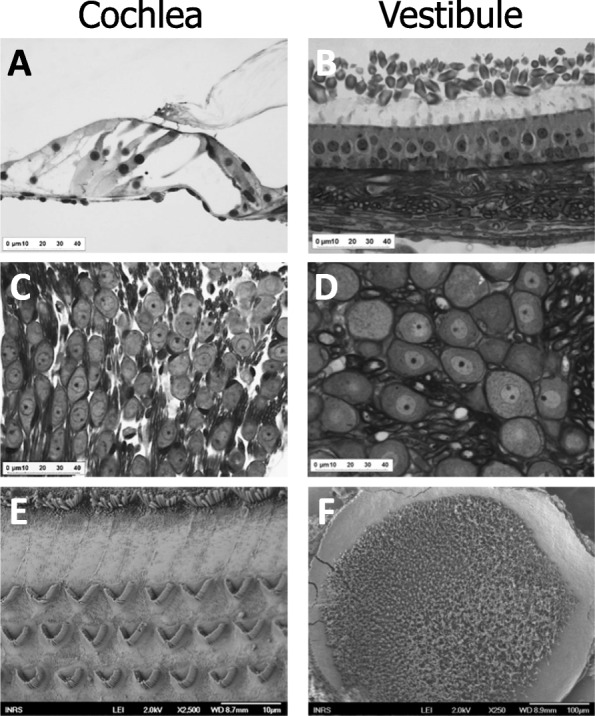
Cochlea (left panels) and vestibule (right panels) were undamaged following co-exposure to noise and 1 × 15 min/h 250 ppm-CS_2_. Representative histological images are shown. Organ of Corti (**a**) and epithelium of the utricle (**b**) were harvested at T1. The apical spiral ganglion (**c**) and Scarpa’s ganglion (**d**) were imaged in samples harvested following the recovery period (T2). Cochlear hair cells in the region detecting 8-kHz frequencies (**e**) and the hair cells from the utricule (**f**) were also imaged in samples harvested at T2

The organ of Corti (Fig. 5a), the vestibular epithelium (Fig. 5b), the spiral ganglion (Fig. 5c) and the Scarpa ganglion (Fig. 5d) were observed in semi-thin sections of epoxy-resin-embedded specimens.

Qualitative observations of organ of Corti and vestibular epithelium harvested at T1 revealed no morphological alterations in any of the experimental groups. In the organ of Corti, IHCs, OHCs, Deiters’ cells and Hensen’s cells showed normal morphological features from the basal turn to the most apical region. Moreover, the vestibular epithelium remained morphologically intact regardless of the exposure conditions. Spiral and Scarpa ganglion cells were observed 4 weeks after the end of exposure (T2) and, once again, no alterations were observed.

Finally, auditory and vestibular sensory epithelia from T2 were observed by scanning electron microscopy. Regardless of the group, no evidence of injury to stereociliae was found (Fig. 5e-f), and samples from all treated animals resembled those from control animals. These data demonstrate that neither the low-frequency noise nor the combination of noise and CS_2_ caused injury to stereociliae.

## Discussion

In occupational settings, workers are often exposed to chemicals at very irregular time intervals and in different spaces. Occupational exposure to CS_2_ is closely associated with certain tasks and is therefore very variable during the work shift. Modeling this kind of exposure in animal models is a real challenge for toxicologists because it is technically more complex to reproduce than continuous exposure, and because it adds a variable to the equation linking dose and effect: the variability of the exposure over time.

The duration of the exposure peaks and the interval between peaks are key parameters. If the interval is sufficient to allow the elimination of the majority of the body’s burden of the toxic compound, i.e., the mother molecule or toxic metabolites, the effects of separate peaks remain independent. In the opposite case, a cumulative phenomenon may occur, which could lead to higher concentrations of the toxic compound at the end of the work shift. Although experimental constraints prevented us from testing the influence of the durations and intervals between CS_2_ peaks separately, we can nevertheless present this first comparison between fractioned and continuous exposure to solvent and the effects the different conditions exert on hearing and balance.

The results obtained during the current study confirmed that CS_2_ exerts two different transitory effects on the auditory system [17, 29]. First, it reduces the auditory deficits measured at noise-injured frequencies (3.6, 4.8 and 6 kHz) in co-exposed animals compared to rats exposed to noise alone. These decreases in hearing loss at frequencies below 6 kHz were observed in the two groups exposed to intermittent CS_2_ and in the one exposed to the higher continuous concentration (although the difference was just below significance for the 2 × 15 min/h CS_2_ group). The effect was more pronounced with the 1 × 15 min/h CS_2_ at 250 ppm, which corresponds to 10 times the **French** short-term threshold limit value. Surprisingly, with the equivalent dose administered continuously (63 ppm), the decrease was not significant. Furthermore, no difference in blood levels of CS_2_ or urinary TTCA concentrations was detected.

These results are in agreement with those obtained in a previous study, which showed that co-exposure to CS_2_ at 250 ppm (15 min/h) and a noise centered at 8 kHz for 1 week is less traumatic than exposure to noise alone [29]. Moreover, a similar phenomenon has also been described with other solvents: the combination of continuous noise with 300 ppm styrene also led to lower hearing loss than a continuous noise alone [30, 44].

CS_2_ had no cochleotoxic effect, so this observation may not be the result of a cochlear interaction. It is possible that CS_2_ has a rapid pharmacological impact on the CNS, decreasing the threshold for triggering of the middle ear reflex (MER). As described previously [44], this effect could be due to a change in polarization at the level of the plasma membrane [45], which could alter the threshold for the MER trigger. Indeed toluene, an aromatic solvent, has been shown to modify the voltage-dependent functioning of Ca^2+^ channels involved in the MER [46]. These types of modifications could be the reason why CS_2_, as other classes of solvents such as aromatic solvents, decrease the MER threshold, and may explain why co-exposure to noise and moderate concentrations of solvents yield smaller hearing deficits than exposure to noise alone [30, 44].

In contrast to this apparently protective effect, compared to the effects of noise alone, which were restricted to a window up to 6.3 kHz, co-exposure to 250 ppm CS_2_ plus noise resulted in a broadening of the window of injury to 9.6 kHz. This effect was not seen for groups exposed to lower concentrations of CS_2_, in confirmation of our previous results [17] that had shown a dose-effect response with a threshold at 250 ppm. We therefore hypothesized that the extension of the window of affected frequencies could be due to a central effect, specifically the efferent pathway, which could reversibly alter the function of the peripheral sound receptor.

No morphological changes were found at the level of the inner ear, suggesting that the functional deficits recorded in the present study were not the result of major injury to the organ of Corti or to the spiral ganglion neurons.

When assessing balance, the results of this study showed that CS_2_ at the two highest doses could temporarily alter the PRN, therefore disrupting the vestibular system. More precisely, combined exposure to noise and CS_2_ decreased the number of saccades and reduced the duration of PRN. These effects were dose-dependent and temporary. As discussed in [16], CS_2_ could act at the level of the CNS to temporarily disrupt neurotransmission in the circuit controlling VOR.

In conclusion, most of the observed effects on hearing (broadening of the affected frequency range) and balance (modification of saccade number and duration) following continuous or intermittent exposure to CS_2_ display a dose-effect relationship. In contrast, the effect observed at the MER level was maximal with the lowest intermittent dose of CS_2_ (1 × 15 min/h). Interestingly, the same dose administered in a continuous mode (63 ppm) does not seem to significantly affect the MER. This result suggests that intermittent exposure to CS_2_ might have a more severe impact on the central reflex loop controlling the MER than exposure to a lower, stable concentration.

## Conclusion

In terms of occupational health, it would seem that the European occupational exposure limit of 5 ppm is sufficiently protective as the effects on hearing and balance are only significant at a concentration equivalent to 40 times the OEL.

On the other hand, it apparently takes only 15 min/h exposure to CS_2_ at 250 ppm (10 times the French STEL) to significantly impact the central loop involved in the MER. Therefore, this limit for short-term exposures may be insufficiently protective. Such results suggest once again that co-exposure to physical agents should be considered when determining occupational threshold limits for chemical substances.

## Supplementary information

**Additional file 1: Supplementary Figure 1.** DPOAE variation at **(A)** T1 [(DPOAE1- DPOAE0)_exposed_ – (DPOAE1- DPOAE0)_control_] and **(B)** T2 [(DPOAE2- DPOAE0)_exposed_ – (DPOAE2- DPOAE0)_control_] as a function of the f2 primary for five experimental conditions. Values shown correspond to mean ± sem. Error bars are shown only for 2 of the five experimental groups for sake of Clarity.

## Data Availability

The datasets used and/or analysed during the current study are available from the corresponding author on reasonable request.

## Abbreviations

CNS: Central nervous system
CS_2_: Carbon disulfide
DCM: Dichloromethane
DPOAEs: Distortion Product OtoAcoustic Emissions
IS: International Standart
MEK: Methyl-ethyl-ketone
MER: Middle ear reflex
OEL: Occupational Exposure Limit
PNS: Peripheral Nervous System
PRN: Post-rotatory nystagmus
STEL: Short-term exposure limit
TTCA: 2-thiothiazolidine-4-carboxylic acid
VOR: Vestibulo-ocular reflex
L_EX,8h_: Equivalent continuous noise level calculated over 8 h
