# Supplementary material for: Effects of co-exposure to CS2 and noise on hearing and balance in rats: continuous versus intermittent CS2 exposures
Source: J Occup Med Toxicol. 2020 May 11;15:9. doi: 10.1186/s12995-020-00260-5 (PMC7216478; doi:10.1186/s12995-020-00260-5)
Supplement: Supplementary file 1 — Additional file 1: Supplementary Figure 1. DPOAE variation at (A) T1 [(DPOAE1- DPOAE0)exposed – (DPOAE1- DPOAE0)control] and (B) T2 [(DPOAE2- DPOAE0)exposed – (DPOAE2- DPOAE0)control] as a function of the f2 primary for five experimental conditions. Values shown correspond to mean ± sem. Error bars are shown only for 2 of the five experimental groups for sake of Clarity. [file 12995_2020_260_MOESM1_ESM.docx]

**(A)**

**(B)**

Supplementary Figure 1 : DPOAE variation at **(A)** T1 [(DPOAE1- DPOAE0)_exposed_ – (DPOAE1- DPOAE0)_control_] and **(B)** T2 [(DPOAE2- DPOAE0)_exposed_ – (DPOAE2- DPOAE0)_control_] as a function of the f2 primary for five experimental conditions. Values shown correspond to mean ± sem. Error bars are shown only for 2 of the five experimental groups for sake of clarity.
